# Liposarcoma of the Spermatic Cord: A Rare Entity

**DOI:** 10.1155/2011/572973

**Published:** 2011-07-02

**Authors:** Fazl Qadir Parray, Rayees Ahmad Dar, Nisar Ahmad Chowdri, Arif Hamid, Rayees Ahmed Malik

**Affiliations:** Department of General and Minimal Invasive Surgery, Sher-i-kashmir Institute of Medical Sciences, Soura Srinagar 190011, Jammu and Kashmir, India

## Abstract

Primary malignant tumours of spermatic cord are rare. The liposarcoma of spermatic cord is a rare entity and only a few cases have been reported in the literature. We report a case of forty five-year-old male with huge left inguinoscrotal swelling. Fine needle aspiration cytology (FNAC) of swelling revealed the diagnosis of a liposarcoma. The patient was subjected to radical orchidectomy and wide excision. Histopathological examination (HPE) of the resected specimen reported a well-differentiated liposarcoma of the spermatic cord and confirmed the diagnosis.

## 1. Introduction

Liposarcoma of the spermatic cord is a rare condition, representing approximately 7% of paratesticular sarcomas [1]. Only a few studies have reported this disease entity so far.

## 2. Case

**Figure 1:**
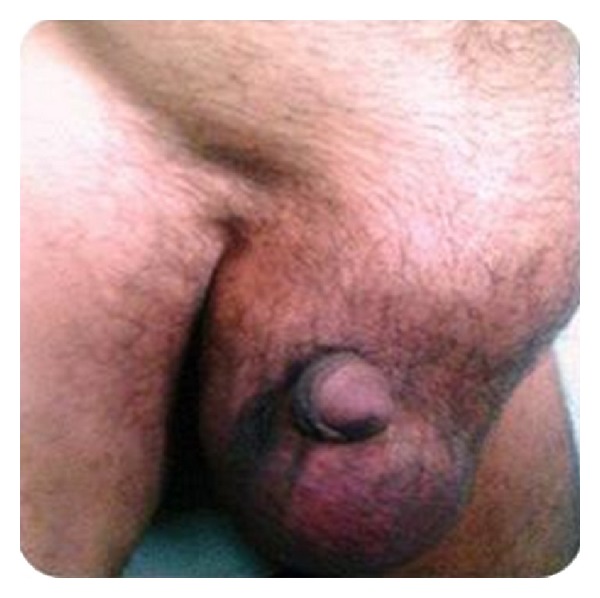
Clinical Photograph of a patient with inguinoscrotal swelling.

A 45-year-old man presented with chief complaint of a progressive, painless swelling in the left inguinoscrotal region from last seven months. Local examination revealed a huge left inguinoscrotal swelling, 11 × 6 cm, with penis pushed to opposite side and almost buried in the swelling (Figure 1).

**Figure 2:**
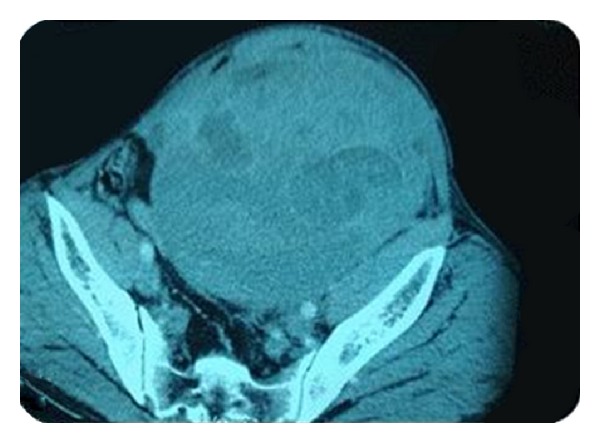
Showing CECT picture of a sarcoma (fat is not delineated nicely).

The swelling had no cough impulse. Palpation revealed a firm, smooth-surfaced tender swelling and at the same time testis could be felt separately. The swelling was irreducible and without any fluctuation. The transillumination test was also negative. Both hernial orifices were competent. There was no associated inguinal lymphadenopathy. Abdominal examination did not reveal any palpable lump. Ultrasonography (USG) reported this mass to be hyperechogenic and inhomogeneous and at the same time separate from the testis and epididymis. FNAC of the swelling was advised, which established the diagnosis of a liposarcoma. Subsequent CECT pelvis and abdomen also revealed a huge paratesticular tumor with an impression of a sarcoma (Figure 2).

**Figure 3:**
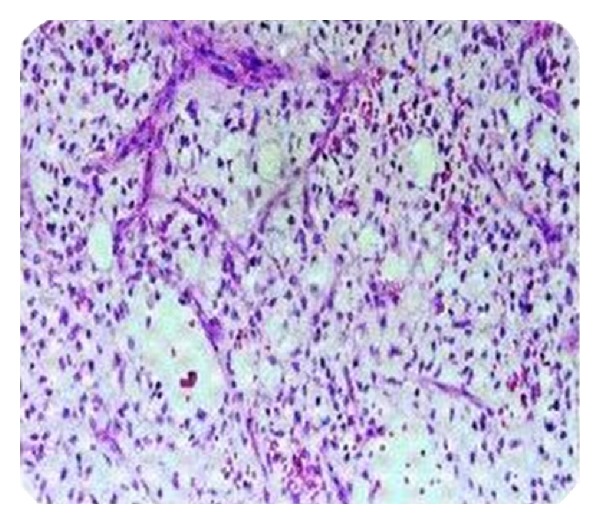
Showing histopathological picture of a well-differentiated liposarcoma.

However, it excluded the presence of any lymph node involvement or distant metastasis. Surgical exploration was carried out with the help of a urologist. On exploration, a mass of lipomatous texture (12 × 5 cm) was found to be arising from inguinal segment of spermatic cord. Radical orchidectomy and wide excision was carried out. Histopathological examination of the resected specimen confirmed diagnosis by revealing a well differentiated liposarcoma (myxoid variant) of the spermatic cord (Figure 3).

The mass did not show any signs of infiltration into the testis or epididymis. All resection margins were found to be free of tumor. Since a complete surgical resection of the tumor with wide margins of 2 cm was achieved so no adjuvant radiotherapy was advised. After 18 months of followup so far the patient does not show evidence of tumour-progression or recurrence and is doing well.

## 3. Discussion

The first case of a spermatic cord sarcoma was reported in 1845 by Lesauvage [2]. 80% of spermatic cord tumors are benign and usually originate from lipomatous tissue. Most paratesticular malignant tumors are usually sarcomas. Lipomatous tissue plays a little role in spermatic cord malignancies, comprising only 5% to 7% of all spermatic cord sarcomas. This tumor is difficult to diagnose preoperatively and is often mistaken for incarcerated hernia, lipoma, or hydrocele. The low grade, well-differentiated and myxoidliposarcomas have favourable prognosis, whereas tumors with multiple recurrences or metastases are likely to be of the high grade, round cell, pleomorphic, or mixed variety.

Liposarcoma is a disease of the older age group. USG provides little information on paratesticular sarcomas, as some are visualized as homogenous and isoechogenic, others as inhomogeneous and echo-density is quite variable. The use of CT scans is not widely reported, but seems to be promising, as liposarcomas are of low density and can be well demarcated, but no pathognomonic features for the differentiation of benign versus malignant masses are defined [3]. Use of MRI provides good information on the local extent, but an exact evaluation of any such mass again cannot be ascertained.

Liposarcomas are locally aggressive tumors and recurrence is quite common after incomplete excision. The inguinal radical orchidectomy with wide resection margin is the standard approach for sarcomas of the spermatic cord [4]. Some authors favour a hemiscrotectomy in addition to the inguinal orchidectomy [5].  Sometimes a second resection is advised if the margins are positive because of the fallacies with frozen section. Local radical excision alone seems to be insufficient for liposarcomas, since local recurrence is a major problem, occurring in up to 50% of the patients [5]. Since a negative resection status can rarely be ensured in the inguinal region, some authors recommend adjuvant radiation.

Routine adjuvant chemotherapy is not justified in liposaroma or any other seminal cord sarcoma due to their resistance against it. Specific outcome data for the disease are not available in the literature. As late recurrence can occur, follow-up examinations should exceed 10 years.

## 4. Conclusion

The primary treatment for the radical cure of the disease continues to be surgery. However, if the margin status is in doubt, adjuvant radiation therapy should be advised. Distant disease has not been reported, but local relapse is common and may occur several years after primary therapy.
